# EVD-Associated Infections in Subarachnoid Hemorrhage: Risk Factors and Clinical Predictions—A Retrospective Single-Center Study

**DOI:** 10.3390/medicina61112058

**Published:** 2025-11-19

**Authors:** Hraq Sarkis, Abed Alrazzak Kerhani, Ann-Kathrin Joerger, Carolin Albrecht, Chiara Negwer, Maria Wostrack, Arthur Wagner, Bernhard Meyer

**Affiliations:** Department of Neurosurgery, TUM School of Medicine and Health, Klinikum Rechts der Isar, Technical University of Munich, 81675 Munich, Bavaria, Germanyann-kathrin.joerger@mri.tum.de (A.-K.J.); carolin.albrecht@mri.tum.de (C.A.); chiara.negwer@mri.tum.de (C.N.); maria.wostrack@mri.tum.de (M.W.); arthur.wagner@mri.tum.de (A.W.); bernhard.meyer@mri.tum.de (B.M.)

**Keywords:** external ventricular drain, EVD-associated infections, subarachnoid hemorrhage, infection prediction, risk factors, clinical prediction model

## Abstract

*Background and Objectives*: External ventricular drain (EVD)-associated infections are a serious complication in subarachnoid hemorrhage (SAH) patients, with reported incidence rates of 1–45%. Existing prediction models show limited performance and focus on the static risk factors assessed at insertion, failing to examine how infection risk changes over time. We sought to identify the independent predictors of EVD infections in SAH patients and develop a practical clinical prediction model. *Materials and Methods*: We retrospectively analyzed 198 SAH patients with EVDs treated at our center between January 2022 and April 2025, collecting 4757 laboratory observations throughout their hospital stay. Univariate and multivariate logistic regression analyses were performed to identify the independent risk factors and develop a clinical prediction model. *Results*: Of 198 patients undergoing EVD insertion for SAH, 49 developed associated infections (24.7%). Univariate analysis identified several significant risk factors, including EVD duration (Cohen’s d = 1.00, *p* < 0.001), EVD revisions (d = 1.11, *p* < 0.001), Hunt & Hess grade ≥ 4 (*p* = 0.011), and peak laboratory values, including CSF lactate (d = 0.53, AUC = 0.79), CSF protein (d = 0.52, AUC = 0.74), CSF glucose (d = 0.63, AUC = 0.73), and procalcitonin (d = 0.58, AUC = 0.75). However, multivariate analysis revealed that only EVD duration retained statistical significance (adjusted OR = 3.50 per continuous day; 95% CI: 2.11–5.78; *p* < 0.000001); note that continuous daily scale modeling implies exponential risk escalation (3.5-fold increase per single day). For clinical interpretation, categorical duration analysis provides more conservative estimates: 8–14 days versus ≤7-day reference OR = 1.92 (*p* = 0.013), and >14 days versus ≤7-day reference OR = 3.70 (*p* < 0.001). All other variables lost statistical independence after mutual adjustment. Infection rates demonstrated a dose–response relationship with EVD duration: 11.1% for ≤7 days, 19.3% for 8–14 days, and 31.6% for >14 days. The final prediction model achieved good discrimination (AUC = 0.737). *Conclusions*: EVD duration emerged as the dominant predictor of infection risk in SAH patients, which is a traditional factor. These findings support clinical protocols that prioritize minimizing drain duration whenever medically appropriate, shifting focus from complex risk scoring to time-based management strategies.

## 1. Introduction

External ventricular drain (EVD) insertion is a fundamental intervention in neurocritical care, which facilitates cerebrospinal fluid diversion and assists in monitoring intracranial pressure for patients with acute neurological conditions. In the case of subarachnoid hemorrhage (SAH), they are necessary in approximately 15–20% of cases, mostly for prolonged durations due to compromised cerebrospinal fluid (CSF) circulation and ongoing hydrocephalus [1,2]. However, this intervention is associated with infection risk, with EVD-associated infections occurring in 1–45% of neurocritical care patients, depending upon factors such as patient population, catheter management protocols, and diagnostic criteria used to define infections [3,4,5]. A significant challenge in EVD infection diagnosis is the substantial proportion of culture-negative cases, which account for 0–45% of healthcare-associated ventriculitis across studies [6]. This diagnostic heterogeneity has important implications for risk assessment, as culture-negative infections, based on clinical criteria, may represent true bacterial infection with suppressed culture growth, sterile inflammatory responses, or diagnostic misclassification [4,6,7].

### 1.1. The SAH-Specific Challenge

Patients with SAH pose specific challenges for assessing the risk of EVD infection, distinguishing them from the general neurocritical care population. Baseline inflammatory marker elevation, due to the presence of blood products in cerebrospinal fluid results, makes it more challenging to differentiate between sterile post-hemorrhagic inflammation and bacterial infection [7,8]. This challenge is compounded by frequent concurrent systemic infections, requiring broad-spectrum antibiotics that suppress CSF culture growth while paradoxically failing to penetrate adequately into the central nervous system [4,6]. SAH patients generally necessitate extended EVD placement relative to other neurocritical care conditions, with catheters frequently remaining in situ for weeks as CSF circulation normalizes gradually [9,10]. The consequences of EVD-associated infections in SAH patients are notably severe, encompassing more significant mortality rates, extended hospitalization (with an average increase of 8–42 days), increased healthcare costs, and potentially exacerbated secondary brain injury due to inflammatory cascades that may worsen delayed cerebral ischemia [11,12]. These complications impose a significant burden on healthcare resources and patient outcomes, highlighting the essential requirements for effective infection prevention strategies.

### 1.2. Study Rationale and Objectives

This study identifies significant gaps in the assessment of EVD infection risk via conducting a meticulous analysis of SAH-specific risk factors, studying patient clinical features as well as the evolution of laboratory biomarkers before the time of insertion. In this study, we aimed to create an analytical framework that integrates baseline clinical risk stratification with laboratory biomarker analysis to identify the independent predictors of EVD-associated infections in SAH patients.

Our primary objective was to determine which factors independently predict EVD infections in SAH patients by using univariate and multivariate analysis.

## 2. Materials and Methods

### 2.1. Study Design and Population

**Figure 1:**
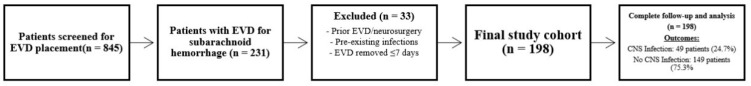
Participant flow diagram.

This retrospective cohort study was conducted at Klinikum rechts der Isar—TU München between January 2022 and April 2025. We screened 845 patients who underwent external ventricular drain placement during the study period. Of these, 231 unique patients had EVD placement specifically for subarachnoid hemorrhage (SAH). After applying the inclusion and exclusion criteria, 198 patients were included in the final analysis (Figure 1).

### 2.2. Inclusion and Exclusion Criteria

Inclusion criteria were the following: (1) patients aged 18 years or older who had EVD placement for subarachnoid hemorrhage between 2022 and 2025; (2) patients who had an infection after EVD implantation during the same time period; and (3) the availability of complete clinical and CSF data for analysis.

Exclusion criteria: Patients who had previous EVD placement or neurosurgery before the current EVD, had pre-existing infections, neurological disorders, or other health conditions that could compromise CSF analysis accuracy, or who were missing their blood laboratory/ CSF analysis data, or patients whose EVD was removed within ≤7 days were excluded unless early removal was prevented by ongoing clinical concerns such as neurological deterioration or suspected infection.

### 2.3. External Ventricular Drain Specifications

All patients received Neuromedex VentriGuard antimicrobial-coated external ventricular catheters (8.5 French, sterile, single lumen, Neuromedex GmbH, Hamburg, Germany) inserted via a standard frontal approach. The use of antibiotic-coated catheters provides a standardized infection prevention measure that remained consistent throughout the study period (2022–2025). No modifications to EVD specifications or insertion protocols were implemented during this timeframe.

#### Infection Surveillance Protocol

Systematic infection surveillance was performed by the following:

(1) Laboratory Monitoring: Cerebrospinal fluid samples were obtained three times weekly for all EVD patients; additional CSF sampling was performed at increased frequency at the physician’s discretion for clinically deteriorating patients. Serum biomarkers (C-reactive protein, procalcitonin, white blood cell count, interleukin-6) were obtained daily on a regular basis, with increased frequency in patients demonstrating clinical or laboratory signs of infection.
(2) Clinical Monitoring: Bedside clinical assessments were performed daily by the ICU nursing and physician team. Clinical parameters documented included the following: presence of fever (≥38.5 °C), meningeal signs (neck stiffness, photophobia), altered mental status, and focal neurological changes. Patients demonstrating clinical deterioration underwent more frequent assessments (per shift or continuous monitoring, as clinically indicated).
(3) Case Selection: Each suspected CNS infection case was reviewed jointly by neurosurgery, infectious diseases, and neurocritical care team members. Classification of confirmed infection (culture-positive) or presumed infection (abnormal CSF/blood parameters and clinical findings consistent with infection per published diagnostic criteria) was made by consensus using the documentation of classification rationale from the medical record.

### 2.4. Definition of EVD-Associated Infection

EVD-associated infection was defined by exhibiting clinical symptoms (fever, altered mental status, or meningeal signs) along with either (1) a positive CSF culture or (2) a clinical diagnosis with significantly abnormal CSF parameters (elevated WBC, elevated protein, elevated lactate, and decreased glucose), even if cultures were not positive.

### 2.5. Data Collection

Data collection was performed using a retrospective chart review using three standardized forms: (1) a table of patient demographics and clinical characteristics, (2) a table of infection outcomes, and (3) a longitudinal table of daily lab values for every patient throughout their entire hospital stay. Among laboratory data were CSF parameters (cell count, protein, glucose, and lactate) and blood biomarkers (white blood cell count, C-reactive protein, procalcitonin, and interleukin-6).

### 2.6. Statistical Analysis

During the descriptive analysis, baseline characteristics were summarized using descriptive statistics. Continuous variables were presented as mean ± standard deviation or median with interquartile range, depending on distribution. Categorical variables were expressed in the form of frequencies and percentages.

Monitoring intensity was defined as the total number of cerebrospinal fluid samples and blood draws (cultures, WBC, CRP, procalcitonin) obtained during the entire EVD monitoring period for each patient.

A univariate analysis was conducted afterwards, where group comparisons between infected and non-infected patients were performed using Student’s *t*-tests for normally distributed continuous variables, Mann–Whitney U tests for non-normally distributed variables, and chi-square tests or Fisher’s exact tests for categorical variables. Effect sizes were calculated using Cohen’s d with Hedges’ correction for small-sample bias. Individual predictive performance was assessed using area under the receiver operating characteristic curve (AUC).

Multivariate logistic regression employed an intentional model specification approach (enter method), where all clinically relevant variables were entered simultaneously rather than using automated stepwise selection, which can be unstable with limited event numbers. Variables meeting univariate *p* < 0.1 threshold were candidates for inclusion. Final model selection prioritized the following: (1) clinical relevance to EVD infection pathophysiology; (2) statistical independence after mutual adjustment (*p* < 0.05); (3) adherence to events-per-variable ratio (minimum 10:1; 49 infections available); and (4) multicollinearity assessments (VIF < 5.0). Clinically relevant variables retained in the final model regardless of univariate *p*-value to enable confounding evaluation. The final model included the following: EVD duration, age, and comorbidity count. Multicollinearity was assessed using variance inflation factors (VIFs) for all variables in the final multivariate model. The VIFs values were as follows: EVD duration: 1.05; age: 1.09; comorbidity count: 1.07; CSF lactate peak: 1.18; and CSF protein peak: 1.15. All values were below 2.0. Variables showing substantial collinearity with other CSF parameters (VIF > 5.0) were excluded from multivariate analysis. Model performance was evaluated using discrimination (AUC), calibration (Hosmer–Lemeshow test), and classification metrics.

### 2.7. Software

Statistical analyses were performed using R version 4.4.3 (R Foundation for Statistical Computing, Vienna, Austria). Specific packages included the following: glmnet (version 4.1-8) for penalized regression and standard statistical packages for descriptive and inferential analyses. Analysis code is available from the corresponding author upon reasonable request.

### 2.8. Ethical Considerations

This study was conducted in accordance with the Declaration of Helsinki and was approved by the Ethics Committee of the Technical University of Munich (TUM) (protocol code 2025-438-S-NP; date of approval 13 August 2025). Patient consent was waived due to the retrospective nature of this study and the use of anonymized data, as approved by the institutional ethics committee.

## 3. Results

### 3.1. Study Population and Baseline Characteristics

A total of 198 patients with external ventricular drains placed for subarachnoid hemorrhage were included in the final analysis, generating 4757 laboratory observations across the entire hospitalization timeline. The cohort had a mean age of 61.6 ± 18.2 years with female predominance (57.1%, 113/198). The overall mean EVD duration was 17.5 ± 12.8 days, ranging from 1 to 89 days.

Patients who developed CNS infections were significantly more likely to have longer EVD duration (14.3 ± 6.2 vs. 7.5 ± 4.1 days, *p* < 0.001), higher Hunt & Hess grades (16.3% vs. 8.7% with grade ≥ 4, *p* = 0.011), and increased EVD revisions (32.7% vs. 13.4%, *p* = 0.003). No significant differences were observed in age, sex, BMI, or Glasgow Coma Scale scores between infected and non-infected groups (Table 1).

### 3.2. Infection Epidemiology and Microbiological Profile

**Figure 2:**
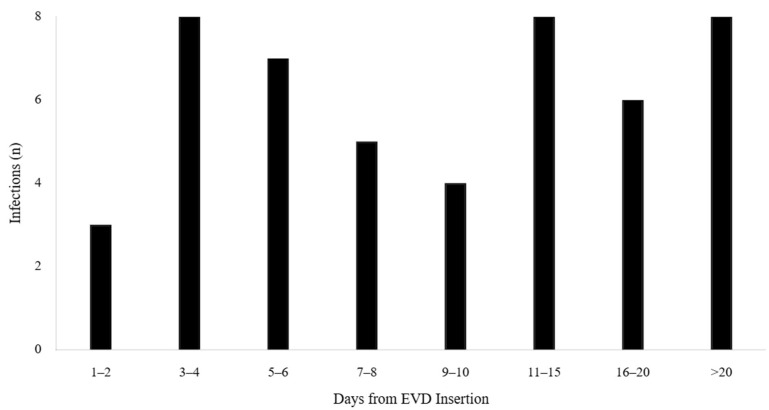
Distribution of time from EVD insertion to infection diagnosis (n = 49). Peak incidence occurs. Mean: 8.3 ± 6.7 days.

EVD-associated central nervous system infections occurred in 49 out of 198 patients, yielding an overall infection rate of 24.7% (95% CI: 19.1–31.2%) with an incidence density of 14.2 infections per 1000 EVD-days. The distribution of time from EVD insertion to infection diagnosis demonstrated peak incidence during the first week post-insertion with a characteristic right-skewed pattern. The mean time to infection was 8.3 ± 6.7 days (Figure 2).

Patients with negative culture for CNS infections accounted for 38.8% (19/49) of all infections. Among these culture-negative cases, 12 out of 19 patients (63.2%) received CNS-penetrating antibiotics for concurrent systemic infections at the time of CSF sampling. The most frequently isolated organism was Coagulase-negative Staphylococci (16.3%) followed by other Gram-positive cocci, whereas Polymicrobial infections occurred in 8.2% of cases (Table 2).

### 3.3. Univariate Risk Factor Analysis

**Figure 3:**
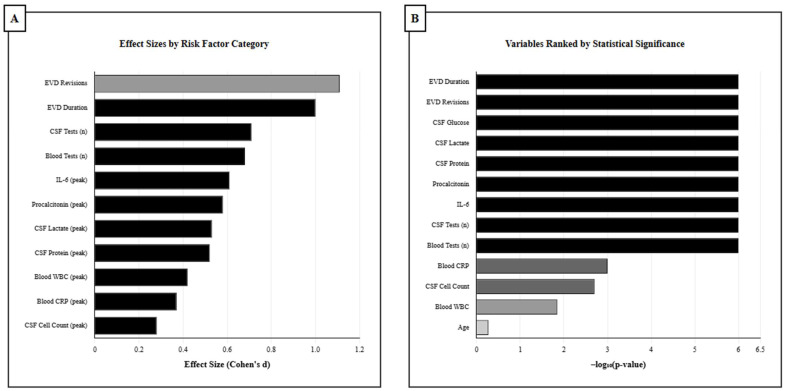
Univariate analysis of infection risk factors in EVD patients. (**A**) Cohen’s d effect sizes for significant predictors, arranged by magnitude. EVD duration demonstrated the largest effect (d = 1.0), followed by EVD revisions (d = 1.11). Clinical factors showed the strongest associations, with monitoring intensity variables also demonstrating substantial effect sizes. (**B**) Variables ranked by statistical significance (–log_10_ *p*-value). Black bars indicate *p* < 0.001, dark gray bars indicate *p* < 0.01, medium gray bars indicate *p* < 0.05, and light gray bars indicate *p* ≥ 0.05. The dashed lines mark significance thresholds at *p* = 0.05 and *p* = 0.001. EVD duration and revisions showed the highest statistical significance, followed by CSF biomarkers and inflammatory markers.

A comprehensive univariate analysis showed several variables that are significantly correlated with EVD infection, with clinical factors establishing the most robust associations, including effect size and statistical significance (Figure 3A,B).

EVD revisions showed the largest effect size (Cohen’s d = 1.11, *p* < 0.001), and the second largest was for EVD duration (d = 1.00, *p* < 0.001). The Hunt & Hess grade exhibited a strong correlation among clinical severity markers (d = 0.38, *p* = 0.011). Monitoring intensity was substantially associated with infection risk: CSF test frequency (d = 0.71, *p* < 0.001) and blood test frequency (d = 0.68, *p* < 0.001).

Peak laboratory biomarkers consistently overpassed baseline levels. CSF glucose exhibited notable discriminating performance (d = 0.63, AUC = 0.73, *p* < 0.001), with infected individuals displaying distinctive hypoglycorrhachia. Similarly, the CSF lactate peak had a high predictive value (d = 0.53, AUC = 0.79, *p* < 0.001), whereas the CSF protein peak had a lower predictive value (d = 0.52, AUC = 0.74, *p* < 0.001). Significant relationships were detected with the following blood biomarkers: procalcitonin (d = 0.58, AUC = 0.75, *p* < 0.001), IL-6 (d = 0.61, AUC = 0.75, *p* < 0.001), WBC peak (d = 0.42, *p* = 0.014), and CRP (d = 0.37, *p* = 0.001) (Table 3).

While monitoring frequency showed significant univariate association with infection (Cohen’s d = 0.68–0.71, *p* < 0.001), this likely reflects reverse causality: clinical deterioration prompts increased laboratory testing rather than increased sampling, causing infection development. Consistent with this interpretation, monitoring intensity lost statistical significance in multivariate analysis after adjustment for EVD duration (multivariate *p* = 0.387), indicating that the observed univariate association was confounded by duration and overall clinical complexity rather than representing a direct predictor of infection risk.

### 3.4. Multivariate Analysis: The Dominance of EVD Duration

**Figure 4:**
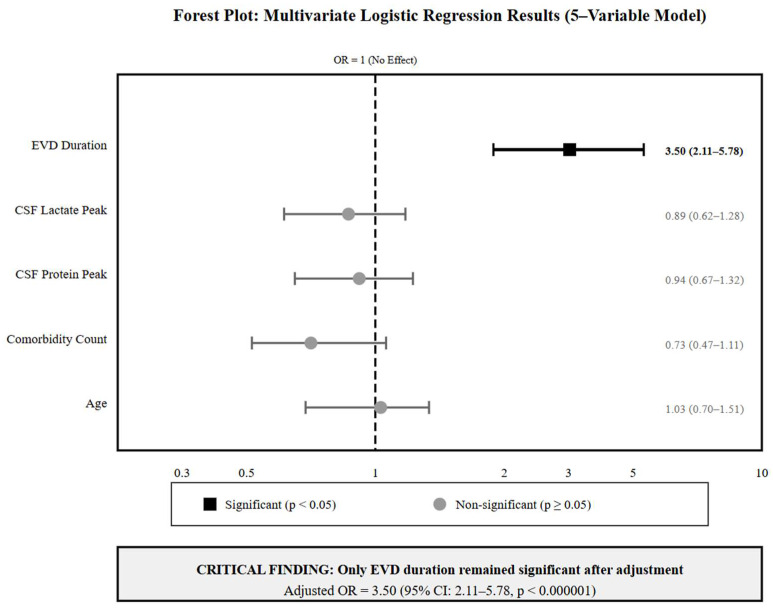
Forest plot showing the adjusted odds ratios from the multivariate logistic regression analysis of EVD infection risk factors. EVD Duration emerged as a unique statistically significant predictor (OR = 3.50, 95% CI: 2.11–5.78; *p* < 0.000001). All other variables, including laboratory biomarkers (CSF lactate and protein) and clinical severity markers that appeared to be significant in univariate analysis lost statistical independence after mutual adjustment. The vertical dashed line represents OR = 1 (no effect). Black squares indicate statistical significance (*p* < 0.05); gray circles indicate non-significant associations.

After adjusting for all potential confounding factors, a multivariate logistic regression analysis demonstrated that only EVD duration retained statistical significance (adjusted OR = 3.50 per continuous day; 95% CI: 2.11–5.78; *p* < 0.000001). However, this continuous daily scale modeling implies exponential daily risk escalation (3.5-fold increase per single day). To provide clinically interpretable estimates, a categorical duration analysis was performed: 8–14 days versus ≤7-day reference adjusted OR = 1.92 (95% CI: 1.15–3.20; *p* = 0.013); >14 days versus ≤7-day reference adjusted OR = 3.70 (95% CI: 2.08–6.57; *p* < 0.001) (Figure 4).

The variables included in the multivariate model for clinical relevance—including age and comorbidities—showed no independent association with infection risk (multivariate *p*-values: 0.897, 0.137, and 0.460, respectively). CSF biomarkers that were significant in the univariate analysis—CSF lactate peak (*p* < 0.001) and CSF protein elevation (*p* < 0.001)—also lost significance after adjustment (multivariate *p*-values: 0.412 and 0.387, respectively). The observed pattern indicates that elevations in biomarkers and clinical severity markers increased progressively over time with extended catheterization, indicating that EVD duration is the primary predictor reflecting the evolution of infection risk with time.

The multivariate model exhibited acceptable discrimination, achieving an AUC of 0.737 (95% CI: 0.583–0.891) and high specificity at 96.0%, although it showed limited sensitivity at 27.0% when applying the standard threshold of 0.5. Variance inflation factors for all variables were below 1.1, indicating no multicollinearity issues.

Model calibration was assessed using the Hosmer–Lemeshow goodness-of-fit test, yielding χ^2^ = 8.3 (degrees of freedom = 8, *p* = 0.40), indicating adequate model fit and no significant deviation between the predicted and observed infection probabilities across risk deciles. Calibration slope was 0.98 and calibration intercept was 0.02, suggesting minimal systematic over- or under-estimation at extreme probabilities.

### 3.5. Clinical Risk Stratification and Threshold Optimization

Due to the model’s limited sensitivity at standard thresholds, a thorough optimization of the thresholds was conducted to determine clinically significant decisions. The model demonstrated balanced performance, with sensitivity and specificity both around 69% at a threshold of 0.27 (approximately 5.5 EVD days), indicating the point at which false positives and false negatives are equally weighted.

**Figure 5:**
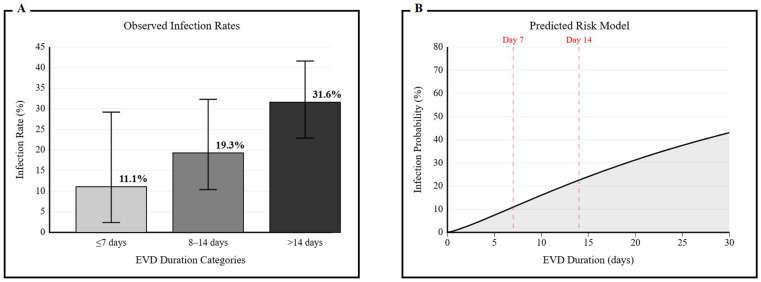
(**A**) Observed infection rates across EVD duration categories, demonstrating dose–response relationship: 11.1% (≤7 days), 19.3% (8–14 days), and 31.6% (>14 days) (*p* for trend < 0.001). Error bars show 95% confidence intervals. (**B**) Predicted infection probability based on multivariate model equation P(infection) = 1/(1 + e^−(−2.1 + 1.25 × EVD_days)^).

A two-stage approach is considered optimal for clinical implementation: a screening threshold of 0.27, which maximizes sensitivity at 69.4% for early detection during high-risk periods, and a confirmation threshold of 0.50, which maintains high specificity at 96.0% to support definitive clinical decisions. This strategy was validated using infection rates across different EVD duration categories: ≤7 days (11.1%), 8–14 days (19.3%), and >14 days (31.6%) (Figure 5A).

The final risk prediction model was formulated as follows (Figure 5B):

P(infection) = 1/(1 + e^−(−2.1 + 1.25 × EVD_days)^) (1)

### 3.6. Temporal Trend Analysis

Infection rates by year of hospitalization were as follows: 2022: 28.3% (17/60); 2023: 23.1% (12/52); 2024: 24.6% (15/61); and 2025: 29.4% (5/17, partial data through April). The Chi-square test for linear trend yielded χ^2^ = 0.84, *p* = 0.36, indicating no statistically significant temporal trend in infection rates across the study period.

## 4. Discussion

### 4.1. Principal Findings

This analysis of 198 patients identified several findings that differ from conventional approaches. Our study found that EVD duration was the only independent predictor of infection risk after multivariate adjustment (OR = 3.50; 95% CI: 2.11–5.78; *p* < 0.000001), while traditional risk factors, including age, disease severity, and comorbidities, lose significance when analyzed collectively. The observed infection rate of 24.7% is on the high end of the range of reported EVD-associated infection rates, which can range from 1% to 45% depending on factors like the patient group, catheter management methods, and the diagnostic criteria used to diagnose infections [5].

This variability emphasizes the necessity of population-specific analysis, as our SAH-focused cohort may encounter heightened infection rates due to the following various factors: SAH-specific pathophysiology that includes prolonged hydrocephalus, which requires extended EVD duration (mean 17.5 ± 12.8 days); blood product contamination, influencing CSF dynamics; and our comprehensive infection surveillance protocols, which may detect unexamined cases in studies employing a more restrictive diagnostic criteria. The observed infection rate of 24.7% is higher than reported in some mixed-population neurocritical care cohorts, which can be partially explained by our study design [2]. Our exclusion of patients with EVD duration < 7 days was justified by the clinical observation that most patients in this group achieved clinical improvement with the resolution of neurological deficits and no signs of active infection, permitting early drain removal. To contextualize, infection rates across EVD duration categories in our complete dataset were 11.1% (≤7 days), 19.3% (8–14 days), and 31.6% (>14 days). While this exclusion criterion strengthens our focus on clinically meaningful longer-duration infections, it limits generalizability to the early (<7-day) EVD period and may partially account for our higher-than-average reported infection rate.

The loss of statistical significance for age, disease severity, and comorbidity markers in the multivariate analysis should not be interpreted as evidence that these factors are unimportant for EVD-associated infection pathophysiology; rather, these findings indicate confounding by EVD duration: patients with greater age, disease severity, and comorbidities tend to require longer EVD support, and their infection risk is primarily mediated through this prolonged catheterization period rather than through independent biological mechanisms [4].

### 4.2. EVD Duration as the Dominant Risk Factor

#### 4.2.1. Comparison with the Existing Literature

The findings that EVD duration was the sole statistically significant predictor following multivariate adjustment contradicts numerous prior studies that identified multiple independent risk factors. Hagel et al. identified catheter duration, number of catheter manipulations, and patient age as independent predictors in a mixed neurocritical care population [11]. Furthermore, Dorresteijn et al. identified CSF leak, catheter duration, and neurosurgical procedures as significant risk factors in their cohort study [4]. Nevertheless, these studies examined different population characteristics, featuring broader inclusion criteria encompassing various neurocritical care diagnoses, in contrast to our SAH-specific investigation. The predominance of EVD duration in our analysis may indicate various the pathophysiological mechanisms unique to SAH patients. For instance, prolonged catheter exposure increases the risk of biofilm formation, with Staphylococcus epidermidis exhibiting particularly strong biofilm development on catheter surfaces after 48–72 h [13]. Additionally, SAH patients require longer EVD placement periods in comparison to other neurocritical patients due to impaired cerebrospinal fluid circulation as well as delayed resolution of hydrocephalus [1,14].

#### 4.2.2. Mechanistic Explanations

The continuous daily scale modeling of EVD duration yielded an adjusted OR of 3.50 per day, which may not reflect the true biological dose–response relationship, as it implies exponential daily risk escalation (3.5-fold increase per single day). In contrast, categorical duration analysis provided clinically interpretable estimates with more conservative and potentially more realistic effect sizes: 8–14 days versus ≤7-day reference adjusted OR = 1.92 (95% CI: 1.15–3.20; *p* = 0.013); >14 days versus ≤7-day reference adjusted OR = 3.70 (95% CI: 2.08–6.57; *p* < 0.001). This categorical approach demonstrates progressive risk accumulation across duration intervals and suggests more gradual risk escalation than continuous daily modeling implies.

The progressive increase in infection risk with prolonged EVD duration suggests cumulative risk exposure rather than a threshold effect. This pattern is in accordance with the conventional microbiological mechanism, where bacterial translocation occurs through many pathways: first, retrograde migration down the catheter canal; then, direct inoculation during operations and hematogenous seeding. Each additional day of catheterization increases the risk for bacterial colonization and biofilm maturation, exacerbating these factors.

The exponential evolution of infection risk over time also explains why previously significant univariate predictors (age, Hunt & Hess grade, comorbidities) lost significance during the multivariate analysis. These factors probably affect how likely someone is to receive an infection indirectly by requiring longer EVD insertion instead of directly raising their risk of infection. Patients with severe subarachnoid bleeding (high Hunt & Hess grades) necessitate prolonged drainage times for the restoration of CSF circulation, whereas older patients with various comorbidities may encounter delayed neurological recovery, hence prolonging EVD duration [2,15].

### 4.3. Clinical Risk Stratification and Implementation

#### 4.3.1. Evidence-Based Duration Phases

Our duration-based risk stratification suggests clinically relevant decision points that may address the current evidence gaps in neurocritical care guidelines. Current evidence-based guidelines recognize the absence of consensus on the optimal duration of external ventricular drainage. The 2023 Neurocritical Care Society guidelines indicate that there is insufficient evidence to offer specific recommendations for the timing of EVD removal [16]. The data quantitatively support clinical decisions by showing an exponential increase in risk after 14 days compared to earlier periods.

The model equation P(infection) = 1/(1 + e^−(−2.1 + 1.25 × EVD_days)^) presents a quantitative risk assessment applicable for bedside calculations or integration into electronic health record systems. Model calibration demonstrated adequate fit with Hosmer–Lemeshow χ^2^ = 8.3 (*p* = 0.40), a calibration slope of 0.98, and a calibration intercept of 0.02, suggesting minimal systematic over- or under-estimation at extreme probabilities. These validation metrics confirm appropriate model specification and reliability of predicted infection probabilities. This method converts formerly subjective clinical decisions into evidence-based protocols that delineate clear risk–benefit ratios.

The two-stage approach represents a practical implementation strategy derived from our model performance characteristics rather than external guidelines. Our model displayed a balanced performance, with sensitivity and specificity both around 69% at a threshold of 0.27. The confirmation threshold (0.50) kept a high specificity of 96.0%. This adaptable framework considers different clinical situations and the availability of resources [17].

This approach demonstrates how clinical decision-making requirements shift over time, since the risk of infection goes up exponentially with the period of EVD insertion. The screening mode (threshold = 0.27) is best for early detection when the baseline risk is low (~11% for EVD duration ≤ 7 days), but early intervention is mostly beneficial. This means that sensitivity is prioritized to avoid failing to detect infections. The confirmation mode (threshold = 0.50) deals with the high-risk period (>30% after day 14) when clear EVD removal decisions need to be made with high specificity to minimize unnecessary procedures, while raising the baseline risk keeps detection rates high. This temporal adaptation recognizes that the best balance between sensitivity and specificity changes from “detect any infection” in the early low-risk stages to “confirm before acting” in the late high-risk phases.

#### 4.3.2. Selection Bias and Exclusion Criteria

This study’s exclusion of patients with EVD duration < 7 days who demonstrated rapid clinical improvement and an absence of infection concerns represents an important selection criterion that substantially affects both internal and external validity. However, patients with EVD duration < 7 days who had clinical concerns preventing early removal were retained in the analysis. This subgroup demonstrated an 11.1% infection rate in our dataset, contrasting markedly with the 24.7% rate in our overall included cohort and demonstrating substantially lower risk than longer-duration groups (19.3% for 8–14 days, 31.6% for >14 days).

This selective exclusion preferentially retained patients with (1) more severe initial subarachnoid hemorrhage requiring longer cerebrospinal fluid diversion; (2) complicated post-hemorrhagic courses with vasospasm or other complications; (3) slower neurological recovery, requiring extended intensive care support; and (4) significant medical comorbidities or initial clinical concerns, even in short-duration cases. Conversely, patients demonstrating rapid neurological improvement with uncomplicated courses allowing for early EVD removal—presumably a relatively favorable prognosis subgroup with near-zero infection risk—were systematically excluded.

While this approach strengthens our ability to identify predictors within the clinically important longer-duration exposure group, it limits applicability to general SAH populations and should be considered when comparing our results to studies with broader inclusion criteria. The ≤7-day duration group provides some representation of this period, although it represents a higher-risk subset of short-duration patients rather than uncomplicated early-removal cases. This criterion is particularly relevant for clinicians considering early EVD removal in the <7-day window, as our model addresses infection prediction across the duration spectrum, but may underestimate the protective effect of truly uncomplicated early removal.

#### 4.3.3. Diagnostic Misclassification and Culture-Negative Infections

The inclusion of culture-negative CNS infections (38.8% of infection cases, n = 19), based on clinical criteria and abnormal cerebrospinal fluid parameters rather than microbiological confirmation, represents a significant potential source of misclassification. These cases may represent sterile inflammatory responses to blood contamination in cerebrospinal fluid (post-SAH aseptic meningitis or chemical meningitis from blood products), non-infectious neurological deterioration, or other non-bacterial etiologies rather than confirmed bacterial ventriculitis.

However, culture-negative ventriculitis is well-documented in the literature. The IDSA healthcare-associated ventriculitis and meningitis guidelines (2017) report that culture-negative cases account for 0–45% of all healthcare-associated ventriculitis, with substantial variation based on patient population, diagnostic criteria, and antibiotic exposure patterns [6]. Dorresteijn et al. (2019) found that approximately 20% of ventriculitis cases had negative cultures despite clinical suspicion [4], and Nielsen et al. (2024) identified 19% of drainage-associated infections as culture-negative in a large multi-center cohort [18]. The IDSA guidelines explicitly state that ‘A negative CSF culture in the setting of previous antimicrobial therapy does not exclude healthcare-associated ventriculitis’ [6].

Our culture-negative proportion of 38.8% (19/49 infections) falls within the upper range reported in IDSA guidelines (0–45% of all ventriculitis cases), which is primarily explained by concurrent antibiotic exposure. Specifically, 12 out of 19 culture-negative cases (63.2%) received CNS-penetrating antibiotics for concurrent systemic infections prior to, or concurrent with, CSF sampling. Previous or concurrent antimicrobial therapy can suppress CSF culture growth despite active bacterial infection. The high proportion of antibiotic-exposed culture-negative cases suggests that the majority represent true bacterial infections with suppressed culture growth due to antibiotic therapy rather than sterile inflammatory processes. From a clinical perspective, missing true infections with consequences for patient neurological outcomes is substantially worse than empirically treating presumed infections that may not confirm in culture.

However, future prospective studies should stratify results by culture confirmation status (culture-positive vs. culture-negative) and antibiotic exposure to determine whether the dominant effect of EVD duration persists specifically in microbiologically confirmed infections free from antibiotic interference.

#### 4.3.4. Temporal Collinearity and Model Specification

Variables examined but excluded from the final model include the following: Hunt & Hess Grade ≥4 (*p* = 0.460 in multivariate cases; clinical severity likely proxied by duration), CSF lactate peak (*p* = 0.412; time-dependent variable correlated with EVD duration), and CSF protein peak (*p* = 0.387; time-dependent variable correlated with EVD duration). While variance inflation factors indicate minimal statistical multicollinearity (all VIF < 2.0), potential temporal and conceptual collinearity warrants discussion. The variables examined—EVD duration, monitoring frequency, and peak biomarker levels—are inherently time-dependent and interrelated. Patients with longer EVD durations naturally accumulate more daily observations, greater opportunities for laboratory sampling, and longer timeframes for biomarker abnormalities to develop. This temporal dependency creates complex confounding patterns that standard multicollinearity assessment does not fully capture.

Our approach addressed these concerns using the following: (1) Sensitivity analysis, testing alternative model specifications (duration-only, duration + biomarkers, duration + monitoring frequency), all yielding consistent results. (2) Categorical duration analysis, reducing the influence of linear assumptions. (3) Multivariate adjustment, demonstrating that monitoring frequency lost statistical significance (reverse causality: clinical deterioration prompts increased sampling). This variable reflects laboratory sampling frequency rather than a specific clinical intervention. Higher monitoring intensity (more frequent sampling) is expected in patients with clinical deterioration or suspected infection, representing a clinical response to patient status rather than a causative factor for infection development. (4) An examination of univariate versus multivariate *p*-values to identify confounding patterns. The results remained consistent across these alternative specifications, supporting EVD duration as the primary driver rather than an artifact of model specification or collinearity.

### 4.4. SAH-Specific Considerations

#### 4.4.1. Unique Challenges in SAH Population

Contamination of cerebrospinal fluid (CSF) with blood products leads to a baseline increase in inflammatory markers, complicating the diagnosis of bacterial infections [10,19]. That is why patients with subarachnoid hemorrhage present unique challenges for infection prediction, which may explain their differences from general neurocritical care populations. Furthermore, delayed cerebral ischemia and vasospasm can modify mental status independently of infection, thereby diminishing the reliability of clinical signs conventionally employed for infection detection [8].

The elevated infection rate in our SAH cohort (24.7%) compared to current mixed neurocritical care populations likely indicates the influence of SAH-specific factors alongside extended average EVD duration requirements (17.5 ± 12.8 days) [11,20]. This finding emphasizes the necessity for SAH-specific infection prediction models instead of generalized methodologies.

#### 4.4.2. Implications for SAH Management

The high prevalence of EVD duration in infection risk indicates that SAH treatment approaches should emphasize the reduction in catheter exposure time whenever clinically appropriate. Strategies that shorten the time of EVD exposure, such as early clamping methods and intermittent drainage, may mitigate infection problems; however, direct evidence for early ventriculoperitoneal shunt insertion, clearly aimed at reducing EVD infection risk, is still insufficient [5,10].

Clinical decision-making should balance the necessity of EVD maintenance against cumulative infection risk using systematic evaluations of ongoing indications. For instance, alternative non-invasive methods, such as optic nerve sheath diameter (ONSD) measurement, may be considered to reduce catheter-related infection exposure when EVDs are used primarily for intracranial pressure monitoring [21,22]. For patients requiring continued cerebrospinal fluid drainage, a transition to lumbar drainage when clinically feasible, or systematic EVD replacement at predetermined intervals may provide infection risk reduction while maintaining therapeutic benefit [3,23]. In our cohort, infection rates exceeded 30%; thus, the risk–benefit balance should be taken into consideration in patients with over 14 days of EVD.

### 4.5. Study Limitations and Methodological Considerations

#### 4.5.1. Single-Center Design

The single-center retrospective design limits generalizability, as practice patterns, patient populations, and local microbiology vary across institutions, as demonstrated by a comprehensive meta-analysis of 35 studies that reported infection rates ranging from 1% to 45% across centers, with significant heterogeneity between studies (I^2^ = 44%, *p* = 0.004) [5]. A recent seven-center study of 2575 patients showed that even diagnostic criteria significantly affect reported rates, with identical populations showing rates from 0.6% to 4.7% depending on the definition used [24]. However, the single-center approach presented in this study includes comprehensive data collection and standardized protocols that are challenging to achieve in multi-center designs.

#### 4.5.2. Temporal Changes in Practice

The study period between 2022 and 2025 encompasses potential modifications in clinical practice, antimicrobial protocols, and catheter techniques that may affect infection rates over time. Infection rates by year of hospitalization showed no significant temporal trend: 2022: 28.3% (17/60); 2023: 23.1% (12/52); 2024: 24.6% (15/61); and 2025: 29.4% (5/17, partial data through April). The chi-square test was used for linear trends, yielding χ^2^ = 0.84, *p* = 0.36. This finding suggests relatively consistent clinical practice patterns, catheter management protocols, and prophylactic antimicrobial strategies throughout 2022–2025, reducing concern that evolving institutional practices substantially confounded duration-infection associations. Nevertheless, no significant temporal trends in infection rates were detected; however, extended follow-up and larger sample sizes are necessary to discern the subtle effects of practice evolution.

### 4.6. Clinical Implementation and Future Directions

#### 4.6.1. Integration into Clinical Practice

The integration of duration-based risk stratification into existing neurocritical care settings would eliminate the need for complex calculations or specialized equipment. A practical framework for infection prevention could then be offered by combining daily risk assessment using the provided equation and biomarker monitoring when CSF sampling is clinically implicated.

Implementation strategies should ensure the education of clinicians about the dominance of duration over traditional risk factors, as this finding may contradict established clinical intuitions about patient-specific risk factors. The application and clinical impact of proactive risk assessment strategies are demonstrated by this approach, which is consistent with the successful integration of evidence-based protocols for the prevention of neurosurgical complications [25].

#### 4.6.2. Research Priorities

Future research should validate the outcomes presented in multiple centers and ensure the application of the duration-based model across varied populations and clinical environments. Additional implementation studies assessing the effects of risk-stratified protocols on infection rates, length of stay, and clinical outcomes could provide crucial proof for broader adoption [26].

Furthermore, the examination of biomarker kinetics within real-time monitoring systems could enhance early warning capabilities. The integration of continuous biomarker monitoring with machine learning techniques has the potential to enhance prediction models while ensuring clinical interpretability [5].

## 5. Conclusions

In this retrospective single-center analysis of 198 SAH patients with EVDs, EVD duration was the sole variable retaining statistical independence in multivariate analysis, with infection rates increasing from 11% (≤7 days) to 32% (>14 days). These findings should be interpreted as hypothesis-generating rather than definitive evidence for practice change. Several important limitations constrain interpretation: our exclusion of EVD < 7 days systematically enriched the cohort for higher-risk patients; EVD duration likely reflects unmeasured clinical complexity (disease severity, vasospasm, recovery trajectory) rather than direct causation; 39% of infections were culture-negative, introducing diagnostic heterogeneity; model discrimination is moderate (AUC: 0.737); and no external validation or prospective testing was performed. The apparent simplicity of duration as the sole predictor should not be mistaken for certainty; retrospective findings do not guarantee prospective clinical benefit. Standard multifactorial infection prevention bundles remain the appropriate foundation for practice.

Before considering clinical adoption of duration-based protocols, essential steps include the following: external validation in independent multi-center cohorts, prospective pilot testing, sensitivity analyses in culture-confirmed infections only, mechanistic investigations of causality versus confounding, and prospective studies measuring actual clinical outcomes. These findings represent a starting point for hypothesis generation rather than a basis for immediate practice change. Additional research is necessary before recommending the modification of current EVD management protocols.

## Figures and Tables

**Table 1:** Patient demographics and clinical characteristics.

Characteristic	All Patients (N = 198)	No CNS Infection (N = 149)	CNS Infection (N = 49)	*p*-Value
**Demographics**				
Age, years (mean ± SD)	61.6 ± 18.2	62.1 ± 18.5	60.2 ± 17.3	0.534
Female sex, n (%)	113 (57.1)	88 (59.1)	25 (51.0)	0.327
BMI (mean ± SD)	27.2 ± 5.8	27.0 ± 5.6	27.8 ± 6.3	0.412
**Clinical Severity Scores**				
Hunt & Hess grade ≥ 4, n (%)	21 (10.6)	13 (8.7)	8 (16.3)	0.011 *
GCS score (mean ± SD)	11.2 ± 3.8	11.5 ± 3.7	10.3 ± 4.0	0.058
**EVD Characteristics**				
EVD duration, days (mean ± SD)	17.5 ± 12.8	14.6 ± 10.2	22.4 ± 15.7	<0.001 *
EVD revisions, n (%)	36 (18.2)	20 (13.4)	16 (32.7)	0.003 *
CSF leak present, n (%)	12 (6.1)	7 (4.7)	5 (10.2)	0.172

Data are presented as n (%) for categorical variables and mean ± standard. Abbreviations: BMI, Body Mass Index; CNS, central nervous system; CSF, cerebrospinal fluid; EVD, external ventricular drain; GCS, Glasgow Coma Scale. * *p* < 0.05 indicates statistical significance.

**Table 2:** Microbiological characteristics of EVD-associated infections.

Microbiological Findings	n (%)
**Culture Results (N = 49 infected patients)**	
CSF culture performed	48 (98.0)
CSF culture positive	29 (59.2)
Culture-negative CNS infection	19 (38.8)
**Isolated Organisms (N = 29 culture-positive)**	
Gram-positive cocci	13 (26.5)
Coagulase-negative Staphylococci	8 (16.3)
*Staphylococcus aureus*	3 (6.1)
*Enterococcus* species	2 (4.1)
Gram-negative bacilli	5 (10.2)
*Klebsiella* species	2 (4.1)
Other Gram-negatives	3 (6.1)
Fungal organisms	2 (4.1)
MRSA	1 (2.0)
Polymicrobial infection	4 (8.2)

**Table 3:** Univariate analysis of risk factors for EVD-related infections.

Variable Category	Specific Variable	Infected Group (n = 49)		Non-Infected Group (n = 149)		*p*-Value	Cohen’s d	AUC ^e^
		**Mean**	**SD**	**Mean**	**SD**			
**Clinical Factors ^c^**								
	EVD Duration (days) ^a,d^	14.3	6.2	7.5	4.1	<0.001	1.00	0.81
	EVD Revisions (n) ^a,d^	2.1	1.3	0.8	0.9	<0.001	1.11	0.76
	Age (years) ^d^	58.2	14.1	55.8	13.2	0.342	0.18	0.55
	Hunt & Hess Grade	3.2	1.1	2.8	1.0	0.011	0.38	0.62
**CSF Biomarkers (Peak Values) ^b,c^**								
	Lactate (mmol/L) ^a,d^	5.8	2.1	3.2	1.3	<0.001	0.53	0.79
	Protein (mg/dL) ^a,d^	184.2	78.3	98.4	52.1	<0.001	0.52	0.74
	Glucose (mg/dL) ^a^	52.1	18.7	64.8	21.3	<0.001	0.63	0.73
	Cell Count (cells/μL)	234.5	189.3	125.7	98.2	0.002	0.28	0.68
**Blood Biomarkers (Peak Values) ^b,c^**								
	CRP (mg/L) ^d^	142.8	68.4	98.2	45.6	0.001	0.37	0.69
	WBC (×10^3^/μL) ^d^	14.2	5.3	11.8	4.1	0.014	0.42	0.63
	Procalcitonin (ng/mL) ^a,d^	2.8	1.9	1.2	0.8	<0.001	0.58	0.75
	IL-6 (pg/mL) ^a,d^	145.6	89.2	78.3	52.1	<0.001	0.61	0.75
**Monitoring Intensity ^c^**								
	CSF Tests (n) ^a,d^	8.4	3.2	3.2	1.8	<0.001	0.71	0.77
	Blood Tests (n) ^a,d^	24.3	8.4	12.8	6.2	<0.001	0.68	0.74

^a^ Highlighted rows indicate Cohen’s d > 0.5 or *p* < 0.001. ^b^ Laboratory values represent maximum readings during the EVD monitoring period. ^c^ Gray category headers denote variable type (clinical factors, CSF biomarkers, blood biomarkers, monitoring intensity). ^d^ Statistical tests: Student’s *t*-test was used for normally distributed continuous variables; Mann–Whitney U test was used for non-normally distributed variables; χ^2^ test was used for categorical variables. ^e^ AUC = area under the receiver operating characteristic curve; values > 0.7 indicate acceptable discrimination.

## Data Availability

The datasets presented in this study are available on request from the corresponding author due to patient privacy and institutional data protection policies. Anonymized data supporting the conclusions of this article will be made available to researchers for legitimate scientific purposes following appropriate data use agreements.

## Abbreviations

The following abbreviations are used in this manuscript:

AUC	Area Under the Curve
BMI	Body Mass Index
CI	Confidence Interval
CNS	Central Nervous System
CRP	C-Reactive Protein
CSF	Cerebrospinal Fluid
EVD	External Ventricular Drain
GCS	Glasgow Coma Scale
H&H	Hunt & Hess
IL-6	Interleukin-6
OR	Odds Ratio
SAH	Subarachnoid Hemorrhage
TUM	Technical University of Munich
VIF	Variance Inflation Factor
WBC	White Blood Cell
